# Contemporary chest drain practice after pulmonary resection: A multicenter registry study of drain strategy, outcomes, and center-level variation

**DOI:** 10.1016/j.xjon.2026.102053

**Published:** 2026-08-13

**Authors:** Akshay J. Patel, Rebecca Weedle, Abdullah Al-Shammari, Jeesoo Choi, Andrea Bille, ESTS Drainology Collaborative, Enrico Ruffini, Francesco Guerrera, Stefano Caffarotti, Miriam Patella, Hazem Fallouh, Paolo Girotti, Rebecca Weedle, Abdullah Al-Shammari, Jeesoo Choi, Akshay Patel, Andrea Bille, Takahiro Mimae, Morihito Okada, Wolfram Karenovics, Benoit Bedat, Nuria Novoa, Monica Casiraghi, Federico Raveglia, Andrea Cara, Francesco Petrella, Kohei Hashimoto, Yukimi Shibuya, Florian Tomaselli, Nicola Monaci, Ilies Bouabdallah, Arthur Streit, Paolo Mendogni, Mario Nosotti, Giuseppe Cardillo, Sara Ricciardi, Vincent Fang, Luca Luzzi, Chiara Catelli, Lukas Veres, Liesbeth Desender, Kei Suzuki

**Affiliations:** a Department of Thoracic Surgery, Guy's Hospital, Guy's and St Thomas' NHS Foundation Trust, London, England, United Kingdom; b College of Medical and Dental Sciences, Institute of Immunology and Immunotherapy, University of Birmingham, Birmingham, England, United Kingdom; c Department of Thoracic Surgery, St James' Hospital, Dublin, Ireland

**Keywords:** chest drain management, chest tube size, digital drainage systems, enhanced recovery after surgery, length of stay, prolonged air leak, pulmonary resection

## Abstract

**Objectives:**

Chest drain management after pulmonary resection varies widely, particularly with respect to drain size, use of digital systems, and suction strategy. Although smaller drains and digital drainage systems are increasingly adopted, robust real-world data evaluating their impact on clinically relevant outcomes are limited. Using a large contemporary multicenter registry, we evaluated associations between chest drain characteristics and postoperative outcomes, and quantified center-level variation in drain practice.

**Methods:**

A prospective multicenter observational registry including 3348 patients undergoing pulmonary resection across 21 international centers was analyzed. Patient, disease, operative, and drain-related variables were recorded. Primary outcome was prolonged air leak (>5 days). Secondary outcomes included pulmonary complications, surgical emphysema, pleural readmission, length of stay, drain duration, and in-hospital mortality. Multivariable regression models adjusted for patient, operative, and center-level factors were constructed. Center-level variation in drain practice and outcomes was examined descriptively and using funnel plots.

**Results:**

The cohort comprised 3348 patients, of whom 88% underwent minimally invasive resection. Prolonged air leak occurred in 9.0%, pulmonary complications in 7%, and in-hospital mortality in 0.9%. Larger drain size was independently associated with lower odds of prolonged air leak (adjusted odds ratio per 1Fr increase ≈1.12), whereas digital drainage systems were associated with higher detected prolonged air leak but shorter length of stay. Substantial center-level variation was observed in digital drain adoption, median drain size, and prolonged air leak rates, exceeding expected random variation in several centers. Reanalysis incorporating 3 additional international centers demonstrated no material change in adjusted effect estimates.

**Conclusions:**

In this large contemporary registry, chest drain strategy was independently associated with postoperative outcomes after pulmonary resection. Marked intercenter variation in drain practice persists, underscoring the need for evidence-based standardization. These real-world data provide a platform to rationalize chest drain management and inform future pragmatic trials.

## Graphical Abstract

**Figure:**
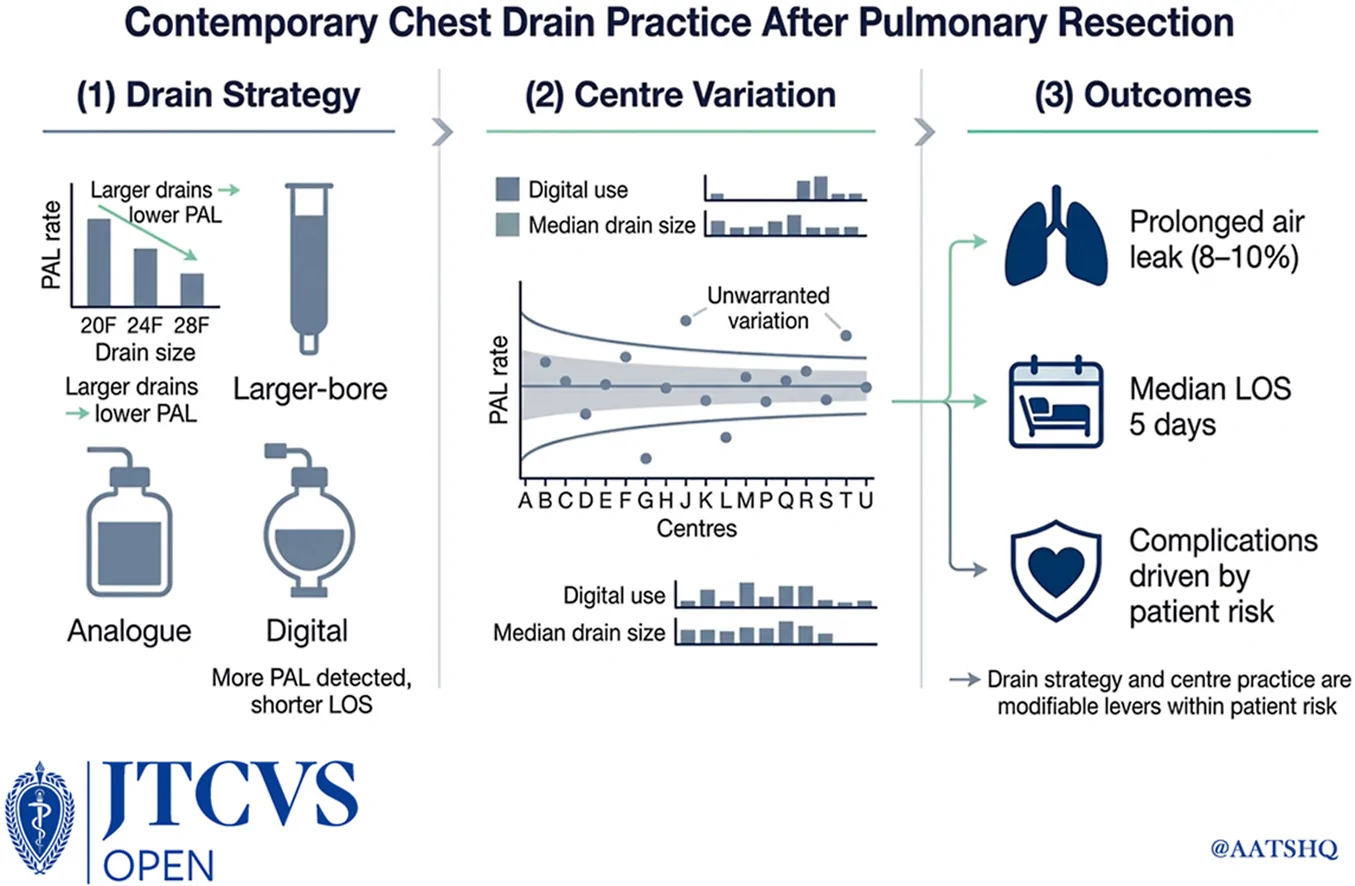

**Figure:**
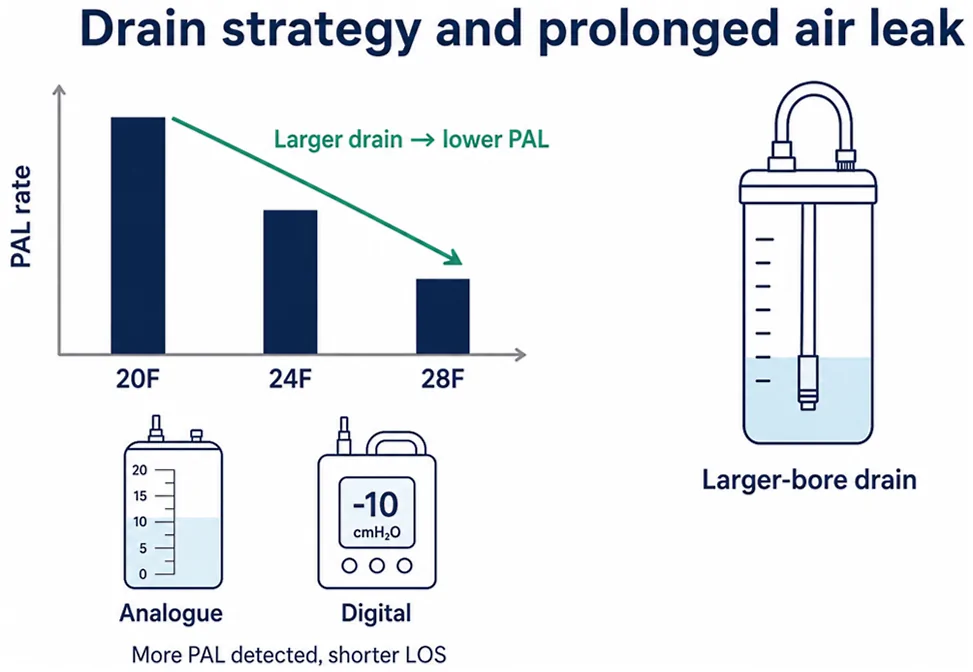
Conceptual summary of how drain strategy and center-level practice influence outcomes.

**Central Message**

Contemporary chest drain strategy and center-level practice independently influence air leak and resource use after pulmonary resection, representing modifiable levers within patient-driven outcomes.

**Perspective**

Multicenter registry data show that drain size, drainage system type, and center practice are associated with PAL and resource use after pulmonary resection. The study challenges assumptions that small-bore drains are benign, clarifies that digital systems increase detection rather than severity, and highlights chest drain management as a modifiable target for standardization and trials.

Chest drainage after pulmonary resection is a core component of perioperative care and directly influences pain, mobilization, pulmonary function, and length of stay.^1^^,^^2^ Despite its central role, chest drain management remains one of the least standardized aspects of thoracic surgical practice, with wide variation in tube size, number of drains, suction strategy, and use of digital drainage systems between centers.^1^^,^^3^^,^^4^ Prolonged air leak (PAL) is the most frequent complication after lung resection and is a major driver of prolonged drainage, delayed discharge, and pleural space complications, as well as increased resource use.^5^, ^6^, ^7^ Within enhanced recovery after surgery (ERAS) pathways, chest tube strategy encompassing drain size, drainage system type, suction application, and explicit removal thresholds has been identified as a key, but insufficiently defined, determinant of postoperative outcomes.

Randomized trials and observational studies suggest that smaller-bore drains and digital drainage systems may improve comfort, standardize decision-making, and reduce length of stay, but most series are single-center, underpowered, or restricted to selected minimally invasive procedures, and they use heterogeneous definitions of PAL and postoperative pulmonary complications.^8^^,^^9^ Large, multicenter datasets are therefore needed to clarify how contemporary chest drain strategies relate to PAL and other clinically relevant outcomes in real-world practice, and to quantify the extent to which center-level practice patterns contribute to variation. In this prospective international registry, we sought to (1) characterize contemporary chest drain practice after pulmonary resection; (2) evaluate associations between drain size, drainage system type (digital vs analogue), suction strategy, and the primary outcome of PAL, alongside secondary outcomes including postoperative pulmonary complications, drain duration, and length of stay; and (3) examine center-level variation in drain practice and outcomes as a platform for future standardization and pragmatic trials.

## Materials and Methods

### Study Design and Setting

This was a prospective, multicenter observational registry study evaluating chest drain practice after pulmonary resection from November 2023 to November 2024. Consecutive patients were enrolled across 21 high-volume thoracic surgery centers in Europe, North America, and Asia. The registry was designed to capture real-world variation in chest drain strategy and associated postoperative outcomes. Local institutional approvals were obtained in accordance with national governance requirements.

### Study Population

Adult patients (≥18 years) undergoing pulmonary resection, including wedge resection, segmentectomy, lobectomy, and bilobectomy, were eligible for inclusion. Patients undergoing pneumonectomy or lung volume reduction surgery or nonpulmonary thoracic procedures (empyema, pleural effusion or hemothorax) were excluded. The final analytic cohort comprised 3348 patients.

### Data Collection

Data were prospectively recorded using a standardized registry template and included patient demographics, comorbidities, operative details, chest drain characteristics, and postoperative outcomes. Patient variables included age, sex, body mass index, smoking status (never, ex-smoker, current smoker), Eastern Cooperative Oncology Group (ECOG) performance status, cardiovascular comorbidity, respiratory comorbidity (chronic obstructive pulmonary disease, asthma, interstitial lung disease, bronchiectasis or previous tuberculosis; disease severity was not coded beyond the available spirometric and gas-transfer measurements), prior thoracic surgery, renal dysfunction, immunosuppression, and preoperative lung function where available. Operative variables included indication for surgery, surgical approach, laterality, extent of resection, lymphadenectomy, intraoperative blood loss, and technical conversion. Chest drain variables included drain size (French), drain type, use of digital versus analogue drainage systems, application of suction, and use of hemostatic or sealant adjuncts. Hemostatic adjuncts and sealants were categorized according to mechanism and composition. Matrix agents included Arista AH (Medafor Inc,; distributed by BD/Bard), SURGICEL Fibrillar (Ethicon, Johnson & Johnson), and HEMOPATCH (Baxter Healthcare SA). Biological agents comprised FLOSEAL (Baxter Healthcare SA), SURGIFLO (Ethicon, Johnson & Johnson), TachoSil (Corza Medical), Beriplast P (CSL Behring GmbH), Bolheal (Chemo-Sero-Therapeutic Research Institute), and BioGlue (CryoLife Inc). Synthetic sealants included polyglycolic acid sheets (eg, Neoveil, Gunze Ltd) and the Veriset hemostatic patch (Medtronic). Sutures were recorded separately as a distinct hemostatic intervention.

### Outcomes

The primary outcome was PAL, defined as an air leak persisting beyond postoperative day 5. Secondary outcomes included postoperative pulmonary complications (PPCs) (composite of pneumonia, acute respiratory distress syndrome, clinically significant atelectasis, when atelectasis was only coded when it prompted active intervention such as bronchoscopy, escalation of respiratory support, or documented treatment beyond routine postoperative physiotherapy), and postoperative bronchopleural fistula), severe surgical emphysema, pleural space complications (pneumothorax, pleural effusion, or empyema), all-cause readmission, unplanned intensive care unit (ICU) admission, return to the operating room, new-onset atrial fibrillation, and other recorded surgical or medical complications including in-hospital mortality. A detailed breakdown of individual complications is provided in Online Data Supplement. Drain duration and length of hospital stay were evaluated, but 90-day mortality was not systematically captured across centers and could not be reliably assessed.

### Statistical Analysis

Continuous variables are reported as median with interquartile range (IQR) and categorical variables as number and percentage. Univariate comparisons were performed using the Wilcoxon rank-sum test for continuous variables and the chi-square or Fisher exact test for categorical variables, as appropriate. Multivariable logistic regression models were constructed for binary outcomes, including prolonged air leak, PPC, pleural readmission, unplanned ICU admission, and in-hospital mortality. Multivariable linear regression models were used for continuous outcomes, including length of stay and drain duration. Covariates entered into the initial models included patient demographics, comorbidities, smoking status, ECOG performance status, operative approach, extent of resection, and chest drain characteristics. Stepwise backward elimination was used to derive final models, retaining only clinically relevant variables and those with statistical significance. Center was included as a fixed effect in all multivariable models to account for institutional practice variation. Effect estimates are presented as odds ratios (ORs) or regression coefficients with 95% CIs.

### Propensity Score Matching

To further address confounding by indication, propensity score–matched analyses were performed for key comparisons, including digital versus analogue drainage systems and drain size strategies. Propensity scores were derived using logistic regression incorporating patient demographics, comorbidities, smoking status, ECOG performance status, operative approach, extent of resection, drain size and center. One-to-one nearest-neighbor matching without replacement was performed using a caliper of 0.2 SDs of the logit of the propensity score as per Austin and colleagues.^10^ A total of 1792 patients from the original cohort were matched according to the drainage system used (digital or underwater seal). Covariate balance was assessed using standardized mean differences. Outcomes were compared between matched groups using appropriate paired statistical tests. Logistic and linear regression modeling were used according to data distribution of the outcome variable with drain type as the exposure, along with covariates and their interaction as predictors. We included full matching weights in the estimation. The comparisons ( ) function in the *marginaleffects* package was used to perform g-computation in the matched sample to estimate the average treatment effect of the treated population. A cluster-robust variance was used to estimate its standard error with matching stratum membership as the clustering variable. All analyses were conducted using R 4.2.3 and the *MatchIt, cobalt, sandwich, lmtest,* and *marginaleffects* packages.^11^, ^12^, ^13^, ^14^

### Center-Level Variation

Center-level variation in drain practice and outcomes was explored descriptively and using funnel plots with 95% and 99.8% control limits. Centers were anonymized and labeled alphabetically. Chest drain removal criteria were not protocolized across centers and were based on local practice integrating clinical status, imaging, and observed air-leak behavior rather than uniform numerical thresholds. In analogue systems, air leak was defined and recorded according to the treating team's judgement based on bubbling in the water seal; for digital drainage systems, any leak recorded above the manufacturer's lower detection threshold was captured in the registry as an air leak, and PAL was defined solely by duration (air leak persisting beyond postoperative day 5) rather than by absolute flow rate. We were unable to distinguish low-flow leaks that may be clinically insignificant from higher-flow leaks within the dataset, and company-specific thresholds or automated algorithms for “clinically relevant” leak detection were not available; as such, it is possible that some patients with low-flow air leaks on digital systems were misclassified as having PAL.

### Missing Data and Software

Analyses were conducted using complete-case analysis for each model. Missing data were excluded. All statistical analyses were performed using R (version 4.2.5).

## Results

### Patient and Operative Characteristics

A total of 3348 patients undergoing pulmonary resection across 21 centers were included in the analysis. Baseline patient characteristics, operative details, chest drain strategies, and postoperative outcomes are summarized in Table 1. The majority of patients were current or former smokers, and comorbidity burden was typical of a contemporary lung cancer surgical population. Online Data Supplement demonstrate a breakdown of the cardiovascular and respiratory comorbidities in the population, with atrial fibrillation and chronic obstructive pulmonary disease being the most predominant. Minimally invasive surgery predominated, with video-assisted thoracoscopic surgery (55.1%) and robotic-assisted thoracoscopic surgery (32.4%) accounting for 88% of resections. PAL more than 5 days occurred in 8.9% of patients. PPCs were observed in 7%, whereas severe surgical emphysema, readmission for pleural complications, and in-hospital mortality were observed less frequently, in 1.3%, 1.7%, and 0.2%, respectively. Online Data Supplement demonstrate the spectrum of complications in the entire cohort as well as the management strategies used to deal with prolonged air leaks, and the hemostatic agents used. Median length of hospital stay was 5 days (IQR, 3-8), and median duration of chest drainage was 3 days (IQR, 1-5). There was no major difference in length of stay or drainage between continental Europe and Asian countries.

**Table 1:** Baseline patient characteristics, operative details, chest drain strategies, and post-operative outcomes

Characteristic	N = 3348∗
Center	
AOU Senese	273 (8.2%)
Bellinzona	89 (2.7%)
Birmingham	417 (12.5%)
Feldkirch	40 (1.2%)
Guys	237 (7.1%)
Hiroshima University Hospital	191 (5.7%)
HUG	156 (4.7%)
HUPH	25 (0.7%)
IEO	402 (12%)
IRCCS San Gerardo dei Tintori	103 (3.1%)
Kyorin University	179 (5.3%)
Linz	76 (2.3%)
Marseille	153 (4.6%)
Nancy	8 (0.2%)
PoliMi	40 (1.2%)
SCF	187 (5.6%)
Shanghai East Hospital	153 (4.6%)
Turin	83 (2.5%)
UHS	105 (3.1%)
UZ Gent	274 (8.2%)
Washington Suzuki	157 (4.7%)
BMI	24.6 (22, 27.8)
Sex	
F	1412 (48%)
M	1510 (52%)
Age	69 (61, 75)
Smoking status	
Current smoker	602 (22%)
Ex-smoker	1349 (48%)
Never smoker	849 (30%)
ECOG PS	
0	1516 (63.6%)
1	762 (32%)
2	87 (3.6%)
3	16 (0.7%)
4	2 (<0.1%)
5	1 (<0.1%)
Cardiovascular comorbidity	1499 (45%)
Respiratory comorbidity	942 (28%)
Cerebrovascular accident	145 (4.3%)
Renal failure	158 (4.7%)
Immunosuppression	109 (3.3%)
Previous chest surgery	287 (8.6%)
Indication for surgery	
Benign	182 (6.3%)
Primary lung cancer	2326 (80.3%)
Pulmonary metastases	390 (13.5%)
Approach	
Open	419 (12.5%)
RATS	1084 (32.4%)
VATS	1842 (55.1%)
Laterality	
Bilateral	5 (0.2%)
Left	1289 (44%)
Right	1632 (55.8%)
Type of resection	
Bilobectomy	45 (1.5%)
Lower lobectomy	521 (17.8%)
Middle lobectomy	154 (5.3%)
Segmentectomy (multiple)	292 (10%)
Segmentectomy (single)	353 (12%)
Upper lobectomy	958 (32.7%)
Wedge	606 (20.7%)
Lymphadenectomy	
Not performed	538 (16.2%)
Sampling	1063 (32%)
Systematic lymphadenectomy	1718 (51.8%)
Conversion	
No	2740 (96.5%)
Yes (bleeding)	28 (1%)
Yes (technical)	72 (2.5%)
Blood loss (mL)	20 (5, 70)
Digital drain	1320 (48.5% of available data with drain)
Drain size	
19Fr	184 (5.1%)
20Fr	378 (11.3%)
24Fr	1014 (30.4%)
26Fr	2 (<0.1%)
28Fr	1425 (42.6%)
30Fr	273 (8.2%)
32Fr	13 (0.4%)
6/8Fr	36 (1.1%)
No drain	15 (0.5%)
Drain type (n = 3333)	
Coated	15 (0.5%)
Not available	576 (17.3%)
PVC	1147 (34.4%)
Silicone	1595 (48.9%)
Progel	277 (6.8%)
Hemostatic adjunct	1478 (44.1%)
Type of adjunct	
Biological agents	304 (14%)
Matrix agents	467 (21.6%)
No material	1165 (53.8%)
Sutures	153 (7%)
Synthetic	78 (3.6%)
Length of stay (d)	5 (3, 7)
Discharged with Heimlich valve	78 (2.3%)
Length of drain duration (d)	2 (1, 4)
Negative pressure (cmH_2_O)	−10.0 (−20.0, −8.0)
Air leak >5 d	297 (8.9%)
Management of PAL	
Blood or chemical pleurodesis	45 (1.3%)
Conservative	272 (8.1%)
EBV	4 (0.1%)
Not applicable	3019 (90.1%)
Reoperation	8 (0.2%)
Talc slurry	4 (0.1%)
Severe surgical emphysema	42 (1.3%)
Drain blockage	3 (0.1%)
Retained effusion	81 (2.4%)
Drain reinsertion for pneumothorax	52 (1.6%)
Drain reinsertion for effusion	18 (0.5%)
Postoperative empyema	15 (0.4%)
Any postoperative complications	388 (11.6%)
Readmission for pneumothorax/effusion/empyema	58 (1.7%)
Postoperative PPCs	234 (7%)
Cardiovascular complications	91 (2.7%)
Unplanned ICU admission	26 (0.8%)
In-hospital mortality	8 (0.2%)

*BMI*, Body mass index; *EBV*, endobronchial valve; *ECOG PS*, Eastern Cooperative Oncology Group Performance Status; *ICU*, intensive care unit; *PAL*, prolonged air leak; *PPC*, postoperative pulmonary complication; *RATS*, robotic-assisted thoracic surgery; *VATS*, video-assisted thoracic surgery.

∗ n (%); Median (Q1, Q3).

### Center-Level Variation in Drain Practice

Substantial center-level variation in chest drain strategy was observed across the 21 participating units, encompassing drain size selection, adoption of digital drainage systems, suction strategies, and thresholds for drain removal. This variation was evident despite broadly comparable case volumes and surgical approaches, underscoring the absence of consensus standards in postoperative chest drain management. Online Data Supplement illustrates the center-level practice patterns and outcomes in more granularity, specifically with regard to drain strategy and early outcomes. Online Data Supplement shows the “center-mapping information.” As illustrated in Figure 1, *A*, use of digital drainage systems varied markedly between centers, ranging from minimal or selective use to near-universal adoption. Across participating centers, pulmonary resections were almost universally managed with a single chest drain; routine use of dual drains was rare and could not be quantified reliably from the registry fields. Drain duration and length of stay are shown in Table 1. The most frequently used drain sizes were 24Fr and 28Fr, with smaller (20Fr) and larger (≥32Fr) drains used less commonly, as illustrated in Figure 1, *B*. Median drain size differed by more than 10Fr between centers (Figure 1, *B*), with some units consistently favoring smaller-bore drains, whereas others routinely used larger-bore chest tubes. These differences were not explained by procedure mix alone and persisted after accounting for surgical approach and extent of resection.

**Figure 1:**
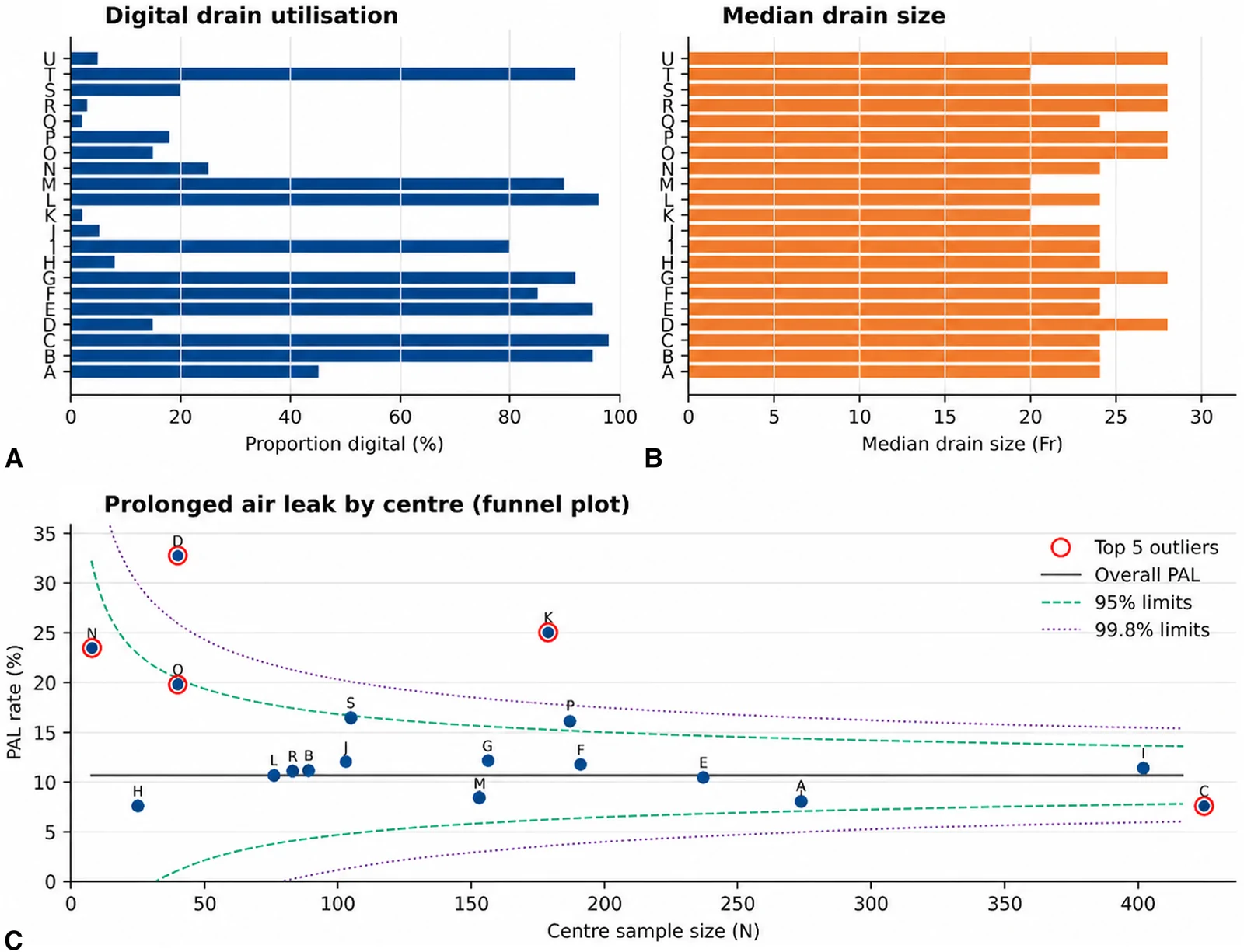
A, Digital drain use bar chart by individual center. B, Median drain size by center. C, Funnel plot to demonstrate the PAL rate by center.

Of note, variation in drain practice was mirrored by variation in clinical outcomes. PAL rates demonstrated wide dispersion across centers, as shown in the funnel plot (Figure 1, *C*). Several centers lay outside the 95% and, in some cases, 99.8% control limits. Centers with high digital drain use tended to report higher rates of PAL but shorter lengths of stay and shorter drainage duration. Conversely, centers that predominantly used larger-bore analogue drains demonstrated lower PAL rates but, in some cases, longer drainage duration. Centers using more 20Fr drains, compared with those that used more 28Fr and 32Fr drains, demonstrated PAL rates of 17% to 25%, 12% to 14%, and 6% to 9%, respectively. Notably, centers favoring smaller drains (<20Fr in size) did not consistently demonstrate superior outcomes in terms of pulmonary complications, readmission, or mortality, despite theoretical advantages related to pain and mobilization. Figure 2 demonstrates the distribution of drain size differences within select centers.

**Figure 2:**
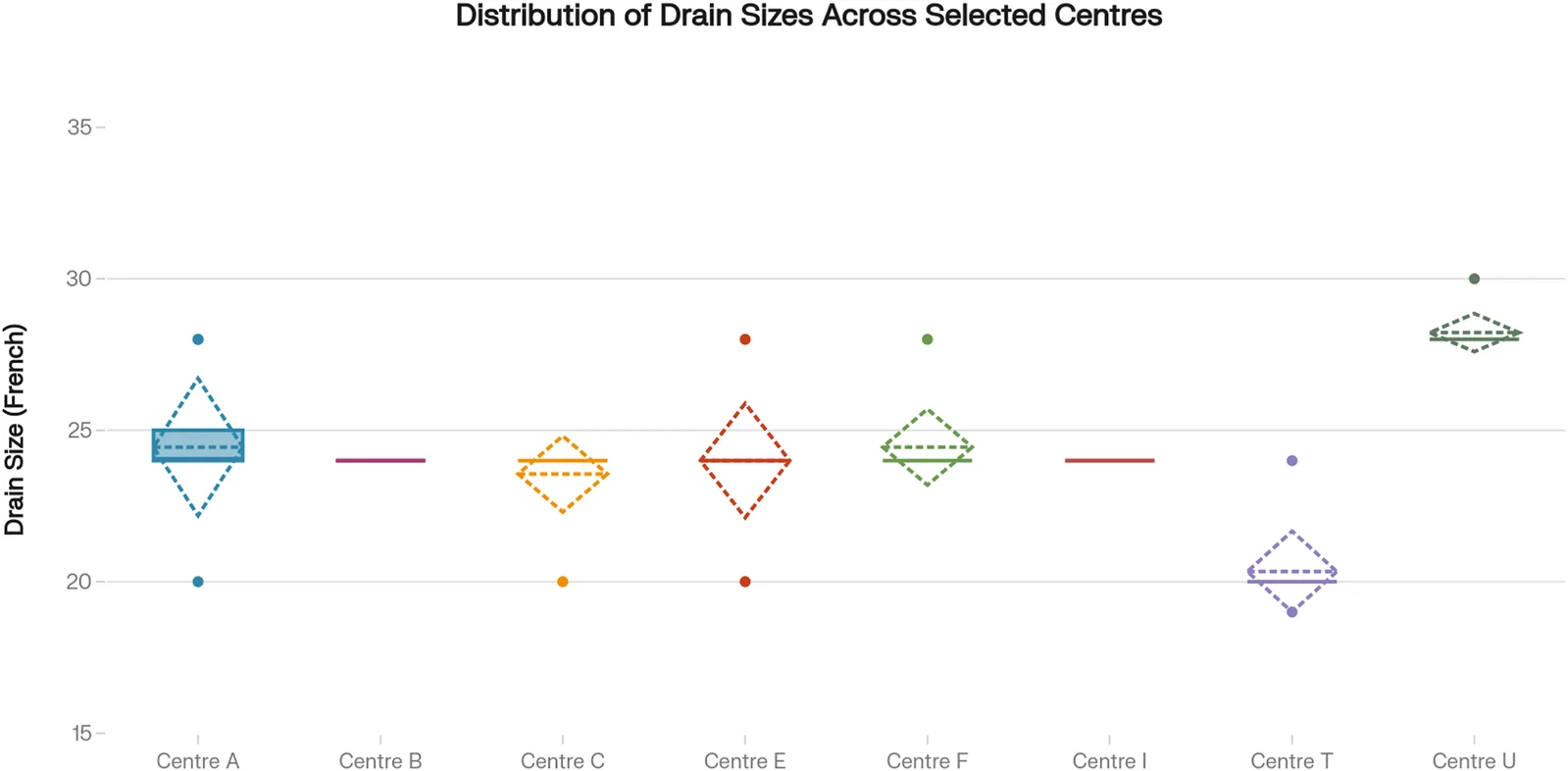
Distribution of drain sizes by selected centers.

### Baseline and Perioperative Characteristics According to Postoperative Pulmonary Complications and In-hospital Mortality

Patients who developed PPCs differed significantly from those without PPC across baseline, operative, and outcome variables. Patients with PPC were more frequently current or ex-smokers (39% and 43% vs 17% and 39%, respectively; *P* < .001) and less commonly never-smokers (17% vs 44%). Cardiovascular (63% vs 46%, *P* < .001) and respiratory comorbidity (49% vs 28%, *P* < .001) were more prevalent. Anatomic resections, particularly upper lobectomy (44% vs 28%), and systematic lymphadenectomy (70% vs 50%, *P* < .001) were correlated, as was conversion (9.2% vs 3.0%, *P* = .003) with PPC. Clinically, PPC was associated with longer length of stay (10 [7-16] vs 5 [3-7] days, *P* < .001), longer drain duration (4.0 [2.0-9.0] vs 3.0 [1.0-5.0] days, *P* < .001), higher rates of PAL (25% vs 7.9%, *P* < .001), unplanned ICU admission (6.4% vs 0.6%, *P* < .001), and in-hospital mortality (3.7% vs 0.1%, *P* < .001).

In-hospital mortality was uncommon (n = 7) but associated with marked differences compared with survivors. All deaths occurred in male patients (100% vs 56%, *P* = .020), who were older (79 [69-81] vs 69 [61-75] years, *P* = .079) and more likely to have ECOG performance status 1 or greater (86%, *P* = .005) and respiratory comorbidity (71% vs 28%, *P* = .023). Nonsurvivors experienced greater blood loss (250 [50-500] vs 100 [20-100] mL, *P* = .056), longer length of stay (7 [6-17] vs 5 [3-8] days, *P* = .040), and longer drain duration (6.0 [2.0-13.0] vs 3.0 [1.0-5.0] days, *P* = .046). In-hospital mortality was strongly associated with postoperative complications (86% vs 7.4%, *P* < .001), PPC (57% vs 3.6%, *P* < .001), and unplanned ICU admission (57% vs 0.7%, *P* < .001).

### Univariate Associations Between Drain Strategy and Outcomes

On univariable analysis, increasing drain size was associated with progressively lower rates of PAL (OR, 0.44, 95% CI, 0.29-0.67; *P* < .0001), demonstrating a clear dose–response relationship across the spectrum of drain sizes used (Figure 3). This inverse relationship persisted on multivariable analysis after adjustment for patient-, operative-, and center-level covariates. Smaller-bore drains were associated with higher rates of PAL and severe surgical emphysema, whereas larger drains were associated with lower observed rates of these complications. Use of digital drainage systems was associated with higher unadjusted rates of PAL than analogue systems (13.0% vs 8.5%), although digital drainage was also associated with shorter length of stay, with no corresponding increase in pulmonary complications, ICU admission, or mortality. By contrast, use of negative-pressure suction was not associated with PAL or other measured outcomes (Online Data Supplement).

**Figure 3:**
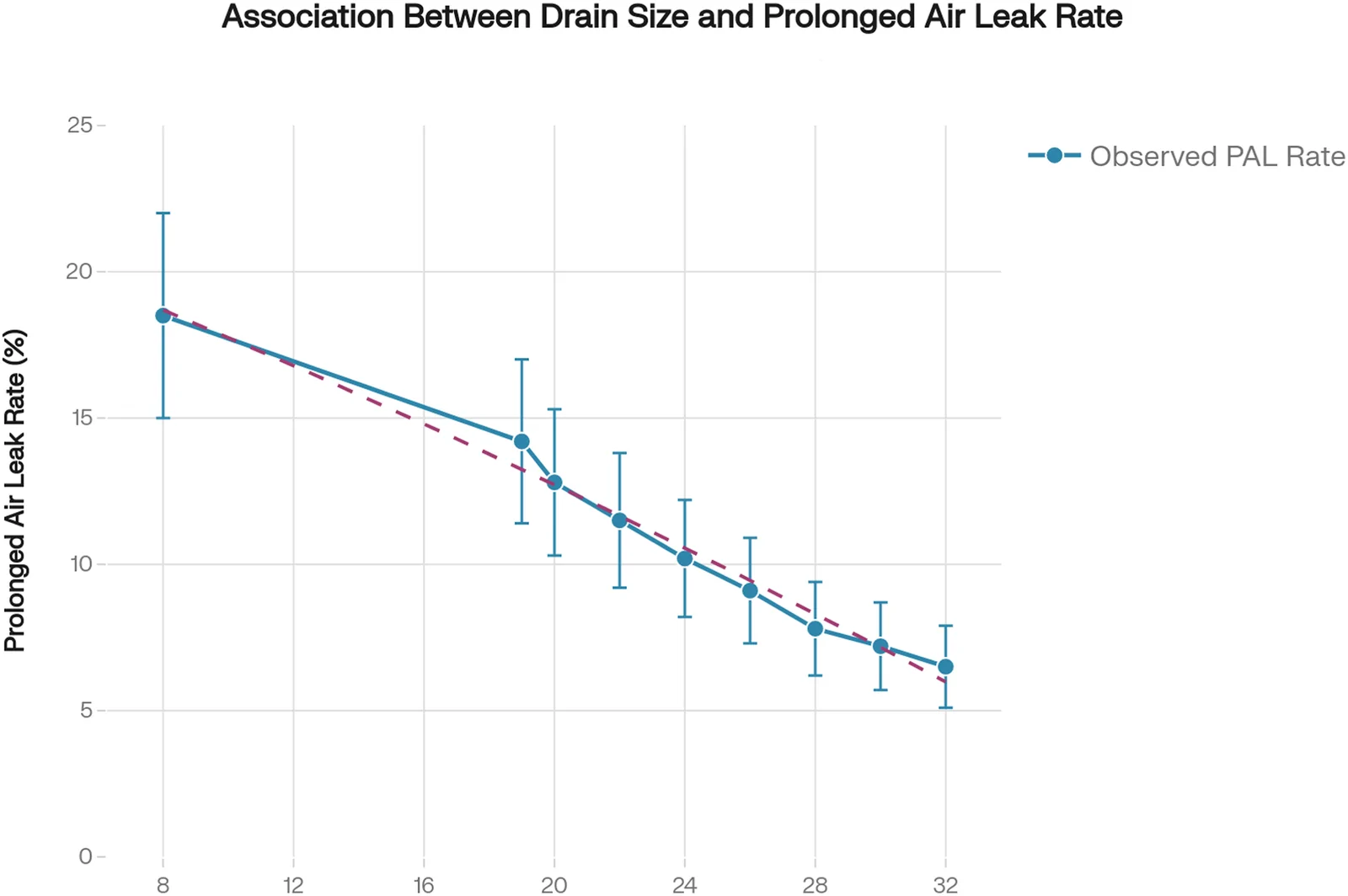
Association between drain size and PAL rate. *PAL*, Prolonged air leak.

Use of hemostatic adjuncts and sealants was common but not clearly associated with a reduction in prolonged air leak. In the dataset, PAL (air leak >5 days) occurred in 11.0% of patients managed without any hemostatic adjunct and in 9.3% of those receiving at least 1 adjunct, with only small absolute differences between groups. When cases were further stratified by adjunct class (matrix agents, biological agents, synthetic sealants or sutures), observed PAL rates ranged from 6.9% to 11.9%, and overlapped with the rate in patients in whom no material was used.

Among patient-level factors, current smoking and respiratory comorbidity showed the strongest unadjusted associations with PAL, with rates of 15.7% in current smokers versus 5.3% in never smokers, and 14.7% in patients with respiratory comorbidity versus 7.8% in those without. Operative factors were also relevant on univariable testing, with PAL rates varying by surgical approach and type of resection, being highest after open surgery and after bilobectomy or upper lobectomy. Overall, patient- and procedure-level characteristics appeared to demonstrate stronger unadjusted associations with PAL, postoperative pulmonary complications, and composite morbidity than most other drain-related variables.

Patients experiencing PAL (>5 days) also demonstrated distinct characteristics. PAL occurred more frequently in older patients (70 [62-76] vs 69 [61-75] years, *P* = .024), current or ex-smokers (32% and 50% vs 17% and 38%; *P* < .001), and those with cardiovascular (56% vs 46%, *P* = .001) or respiratory comorbidity (48% vs 27%, *P* < .001). PAL was more common after anatomic resections, particularly upper lobectomy (44% vs 27%), and less frequent after wedge resection (10% vs 33%). Patients with PAL more often underwent systematic lymphadenectomy (74% vs 49%, *P* < .001) and conversion (6.0% vs 2.0%, *P* < .001). PAL was associated with markedly prolonged length of stay (9 [7-13] vs 5 [3-7] days, *P* < .001), longer drain duration (8.0 [6.0-12.0] vs 2.0 [1.0-4.0] days, *P* < .001), severe surgical emphysema (7.3% vs 0.6%, *P* < .001), pleural readmission (effusion) (4.4% vs 1.4%, *P* = .002), PPC (11% vs 3.1%, *P* < .001), and unplanned ICU admission (4.0% vs 0.6%, *P* < .001). Among patients who desveloped PAL, initial management was conservative in the majority, with adjunctive interventions including blood pleurodesis, talc pleurodesis, reoperation, and endobronchial valves used in a minority of cases, as detailed in Online Data Supplement.

Fluid drainage volumes were also associated with the duration of drainage. Among 1912 patients with complete data, first 24-hour drainage was weakly but significantly correlated with length of drainage (Spearman r = 0.11, *P* < .001), with median duration increasing only modestly from 2 days in the lowest quintile (median 65 mL) to 3 days in the highest quintile (median 475 mL). In contrast, total drainage volume demonstrated a clear dose–response relationship with drain duration (Spearman r = 0.47, *P* < .0001): median duration rose stepwise from 1 day (IQR, 1-3) in the lowest quintile of total output (median 100 mL) to 5 days (IQR, 3-7) in the highest quintile (median 1300 mL) (Online Data Supplement). Drain removal criteria were not protocolized across centers and were not captured as explicit thresholds in the registry; decisions were generally based on local practice, integrating air-leak behavior, imaging, and clinical status rather than a single uniform parameter, yet these data indicate that cumulative drainage volume is more informative than early output alone in predicting how long drains are retained.

### Multivariable Predictors of Prolonged Air Leak

Independent predictors of PAL were identified through multivariable logistic regression with stepwise backward elimination; smoking status was a key determinant of PAL (Table 2). Compared with current smokers, never-smokers had significantly lower odds of PAL (OR, 0.47, 95% CI, 0.31-0.71, *P* = .001), whereas ex-smokers showed no significant difference (OR, 0.81, 95% CI, 0.59-1.13). Respiratory comorbidity was independently associated with increased PAL risk (OR, 1.58, 95% CI, 1.18-2.12, *P* = .036). Procedure-related factors were also important: Wedge resection was independently protective compared with anatomic resections (OR, 0.34, 95% CI, 0.14-0.89, *P* = .004), whereas technical conversion was associated with higher PAL risk (OR, 1.72, 95% CI, 0.87-3.26, *P* = .042). After adjustment for patient, operative, and center-level factors, use of a digital drainage system remained independently associated with higher odds of PAL (OR, 2.05, 95% CI, 1.14-3.67, *P* = .018), consistent with increased detection rather than greater clinical severity. Figure 4 illustrates the unadjusted impact of smoking history, extent of resection, and presence of preexisting respiratory disease on PAL.

**Table 2:** Multivariable regression analysis of predictors for key outcomes

Outcome	Predictor	Effect estimate (OR or β)	95% CI	*P* value
PAL (>5 d)	Never smoker (vs current)	0.47	0.31-0.71	<.001
	Ex-smoker (vs current)	0.81	0.59-1.13	.230
	Respiratory comorbidity	1.58	1.18-2.12	.036
	Wedge resection (vs lobectomy)	0.34	0.14-0.89	.004
	Technical conversion	1.72	0.87-3.26	.042
	Digital drainage system	2.05	1.14-3.67	.018
	Drain size (per 1Fr increase)	0.89	0.85-0.94	<.001
	center effect	Significant	—	<.001
Postoperative pulmonary complications	Never smoker (vs current)	0.46	0.24-0.85	.024
	Ex-smoker (vs current)	0.59	0.36-0.94	.031
	Respiratory comorbidity	2.05	1.32-3.19	.001
	ECOG PS ≥ 1	1.43	1.01-2.01	.044
	Increasing age (per y)	1.06	1.01-1.12	.018
Pleural readmission	Technical conversion	3.10	0.83-9.04	.011
	Drain-related variables	None retained	—	—
Length of stay (d)	Digital drainage system	−0.32	−0.86 to 0.21	.238
	Drain size (per 1Fr increase)	−0.05	−0.12 to 0.02	.180
	Respiratory comorbidity	+1.20	0.45-1.95	.002
	ECOG PS ≥ 1	+0.80	0.21-1.39	.009

*OR*, Odds ratio; *PAL*, prolonged air leak; *BMI*, body mass index; *ECOG PS*, Eastern Cooperative Oncology Group Performance Status.

**Figure 4:**
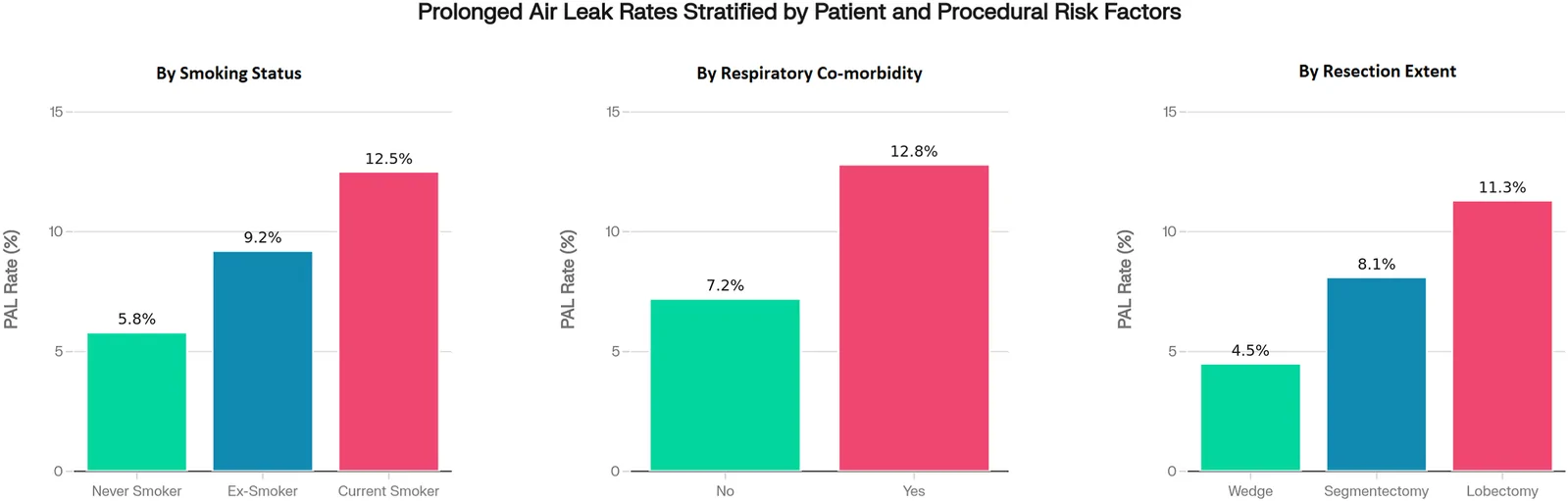
PAL rates stratified by patient and procedural risk factors. *PAL*, Prolonged air leak.

### Pleural Readmission

For pleural readmission due to pneumothorax, effusion, or empyema, technical conversion was the only variable retained in the multivariable model (OR, 3.1, 95% CI, 0.83-9.04, *P* = .011). No drain-related variables, including drain size or drainage system type, were independently associated with pleural readmission after adjustment.

### Composite Postoperative Complications

In multivariable analysis of the composite postoperative complication endpoint, patient factors predominated. Never-smoker status was associated with reduced odds of complications (OR, 0.67, 95% CI, 0.44-1.02, *P* = .041), while increasing ECOG performance status (OR, 1.43, 95% CI, 1.01-2.01, *P* = .044) and respiratory comorbidity (OR, 1.69, 95% CI, 1.22-2.32, *P* = .001) independently increased complication risk. Drain-related variables were not independently associated with composite morbidity after adjustment.

### Postoperative Pulmonary Complications

Smoking status and respiratory comorbidity again emerged as the dominant predictors. Compared with current smokers, both ex-smokers (OR, 0.59, 95% CI, 0.36-0.94) and never-smokers (OR, 0.46, 95% CI, 0.24-0.85, *P* = .024) had significantly lower odds of PPC. Respiratory comorbidity more than doubled the odds of PPC (OR, 2.05, 95% CI, 1.32-3.19, *P* = .001). Drain size, drain type, and use of digital drainage systems were not independently associated with PPC.

### Unplanned Intensive Care Unit Admission and In-Hospital Mortality

Unplanned ICU admission was independently associated with male sex (OR, 2.54, 95% CI, 1.02-7.25, *P* = .046) and increasing age (OR, 1.06 per year, 95% CI, 1.01-1.12, *P* = .018), whereas never-smoker status was protective (OR, 0.51, 95% CI, 0.17-1.38, *P* = .007). Drain-related variables were not retained in the final model. In-hospital mortality was rare; technical conversion was the only variable independently associated with mortality (OR, 4.1, 95% CI, 0.16-54.7, *P* = .045), although CIs were wide due to low event rates.

### Propensity Score–Matched Analyses

Propensity score–matched comparisons of digital versus analogue drainage systems demonstrated that digital drainage was associated with higher observed rates of PAL (OR, 1.58 95% CI, 1.16-2.16, *P* = .004), consistent with multivariable findings, but no increase in PPC, pleural readmission, ICU admission, or in-hospital mortality (Table 3). Digital drainage systems were associated with shorter length of stay (average treatment effect –0.324 days; standard error, 0.274, 95% CI, –0.861 to 0.214, *P* = .238). There was no significant difference in retained effusion (OR, 0.69 95% CI, 0.359-1.33, *P* = .265), drain blockage, drain reinsertion, or postoperative empyema. Propensity-matched analyses examining drain size demonstrated lower rates of PAL and severe surgical emphysema with increasing drain size, without an associated increase in pulmonary complications or mortality, corroborating the primary analyses.

**Table 3:** Propensity score–matched comparison of digital versus analogue drainage systems

Characteristic/outcome	Digital system (n = 896)	Analogue system (n = 896)	Adjusted OR/β (95% CI)	*P* value
Sample size	896	896	—	—
Prematch characteristics				
Age (y), median (IQR)	69 (60-75)	70 (62-76)	—	—
Male sex (%)	55.2%	59.8%	—	—
ECOG PS ≥1 (%)	56.1%	53.2%	—	—
Respiratory comorbidity (%)	27.8%	28.9%	—	—
VATS approach (%)	61.2%	51.4%	—	—
Lobectomy (%)	58.4%	64.2%	—	—
Systematic lymphadenectomy (%)	54.1%	48.6%	—	—
Postmatch characteristics				
Age (y), median (IQR)	68 (60-75)	69 (61-75)	SMD: 0.02	—
Male sex (%)	56.8%	57.1%	SMD: 0.01	—
ECOG PS ≥ 1 (%)	55.4%	54.8%	SMD: 0.01	—
Respiratory comorbidity (%)	28.2%	28.5%	SMD: 0.01	—
VATS approach (%)	56.1%	55.8%	SMD: 0.01	—
Lobectomy (%)	59.2%	60.1%	SMD: 0.02	—
Systematic lymphadenectomy (%)	52.8%	52.3%	SMD: 0.01	—
Primary outcome				
PAL (>5 d)	11.2%	7.4%	1.58 (1.16-2.16)	.004
Secondary outcomes				
Postoperative pulmonary complications	7.1%	7.5%	0.95 (0.65-1.38)	.850
Severe surgical emphysema	1.4%	1.2%	1.18 (0.51-2.72)	.693
Pleural readmission	1.9%	1.4%	0.69 (0.36-1.33)	.265
Drain reinsertion	1.8%	1.5%	1.22 (0.58-2.56)	.600
Retained effusion	2.6%	3.9%	0.65 (0.36-1.17)	.154
Unplanned ICU admission	0.9%	0.8%	1.05 (0.45-2.45)	.901
Length of stay (d), median (IQR)	4 (3-7)	5 (3-7)	−0.32 (−0.86 to 0.21)	.238
Drain duration (d), median (IQR)	2 (1-4)	3 (1-5)	−0.50 (−1.20 to 0.20)	.158
In-hospital mortality	0.2%	0.2%	1.02 (0.14-7.24)	.984

*OR*, Odds ratio; *IQR*, interquartile range; *ECOG PS*, Eastern Cooperative Oncology Group Performance Status; *VATS*, video-assisted thoracoscopic surgery; *PAL*, prolonged air leak; *ICU*, intensive care unit; *SMD*, standardized mean difference.

## Discussion

In this large, prospective, multicenter registry of 3348 patients undergoing pulmonary resection, chest drain strategy was independently associated with key postoperative outcomes, particularly PAL and resource use. Increasing drain size was associated with lower odds of PAL and severe surgical emphysema, whereas use of digital drainage systems was associated with higher detected rates of PAL but shorter length of stay without an increase in pulmonary complications, pleural readmissions, or mortality. Substantial center-level variation was observed in drain size selection, adoption of digital drainage systems, suction use, and thresholds for drain removal, with several centers demonstrating PAL rates beyond 95% and 99.8% control limits, indicating variation beyond that expected by chance alone. Patient- and procedure-related factors, particularly smoking status, respiratory comorbidity, performance status, and extent of resection, remained dominant predictors of postoperative pulmonary complications and composite morbidity. Collectively, these findings support the concept that chest drain management is a modifiable, center-dependent component of perioperative care that interacts with underlying patient risk rather than operating as an isolated determinant of outcome.

Our rates of prolonged air leak, postoperative pulmonary complications, and in-hospital mortality are broadly consistent with contemporary series and reviews of lung resection, although direct comparison is limited by varying definitions of endpoints and differences in case mix.^5^^,^^15^ The median chest drain duration of 3 days and median length of stay of 5 days are aligned with ERAS-oriented studies that emphasize early mobilization and proactive drain removal, despite the observed heterogeneity in drain strategies across centers. Of note, the cohort reflects modern thoracic practice, with a predominance of minimally invasive resections and international representation, enhancing the generalizability of the findings to real-world settings.

The inverse association between drain size and PAL observed in both multivariable and propensity-matched analyses suggests that larger-bore drains may be more effective at preventing or minimizing clinically apparent air leak and severe surgical emphysema. This contrasts with the prevailing assumption that smaller-bore drains are equivalent or superior for air management while conferring advantages in pain and mobilization, an assumption largely extrapolated from small trials and heterogeneous series.^16^ Our data indicate that smaller drains were associated with higher observed rates of PAL and severe surgical emphysema without evidence of reduced pulmonary complications or mortality, challenging the notion that drain miniaturization is universally benign after anatomical resection. Although residual confounding by indication cannot be excluded, the consistency of the association across analytical approaches and its dose–response nature support a potential causal component.

The pattern observed with digital drainage systems, higher detected PAL but shorter length of stay and no increase in clinically important complications, has important implications for how PAL is defined and interpreted in the digital era. Digital systems provide continuous, objective air-leak measurement and likely detect low-grade leaks that may have been missed or subjectively ignored with analogue devices, leading to apparent increases in recorded PAL without greater clinical severity.^6^^,^^8^^,^^9^^,^^17^, ^18^, ^19^, ^20^ The shorter length of stay and lack of signal for increased postoperative pulmonary complications, pleural readmission, or mortality in digital users suggest that these systems facilitate earlier and safe drain removal in appropriately selected patients, even in the presence of ongoing low-volume air leak. These findings support the hypothesis that digital monitoring changes classification rather than risk, and they highlight the need to revisit traditional time-based definitions of PAL to incorporate leak magnitude and clinical impact rather than duration alone.

Our data did not demonstrate an independent association between the initial suction strategy and prolonged air leak, pulmonary complications, or resource use; however, suction status was captured only at the first postoperative time point, and we lacked information on subsequent changes between suction and water seal or adjustments made in response to evolving air-leak behavior and subcutaneous emphysema. In this context, and given the potential for selection bias in the choice and modification of suction, our findings should be considered exploratory and are insufficient to determine whether suction strategy itself is causally associated with outcomes. Current ERAS-informed recommendations discourage routine suction and instead advocate selective use based on intraoperative findings and postoperative physiology,^3^^,^^4^ and our results are broadly compatible with this approach, but we emphasize that drain caliber, adoption of digital monitoring where feasible, and clearer removal thresholds appear more robust targets for standardization than suction strategy per se.

Center-level variation in drain strategy in this registry was substantial, encompassing more than a 10Fr difference in median drain size and a spectrum of digital drain use ranging from minimal or selective use to near-universal adoption. These differences persisted after accounting for surgical approach and extent of resection and were not fully explained by case mix or institutional volume, suggesting that local culture, training, device availability, and risk tolerance are major drivers of practice. Parallel variation in PAL rates, with several centers lying outside the 95% and 99.8% funnel-plot control limits, indicates that these practice patterns translate into clinically relevant differences in outcomes that cannot be due solely to chance. Exploratory comparisons of centers falling above or below the 95% limits did not reveal a single dominant practice pattern that consistently distinguished “high-PAL” from “low-PAL” centers; outliers differed in case mix (including smoking prevalence, respiratory comorbidity, and resection profile) and in drain-related practice (digital vs analogue systems, drain size, and initial suction strategy), with considerable overlap in each characteristic across institutions. Taken together, these observations suggest that center-level differences in PAL are multifactorial, reflecting the combined influence of patient selection, operative complexity, and local chest-drain protocols rather than any single modifiable practice in isolation.

From a health system perspective, such unwarranted variation represents both a quality-of-care concern and a missed opportunity for efficiency. Enhanced recovery frameworks for thoracic surgery explicitly identify chest tube management as a modifiable element of care bundles, yet most guideline documents stop short of prescribing specific drain sizes or technologies because of limited and inconsistent evidence. Our findings provide empirical support for developing standardized, evidence-informed drain pathways that specify preferred drain caliber ranges, criteria for digital system use, and clear thresholds for drain removal, with local adaptation focused on patient risk rather than institutional habit. Benchmarking centers against peers using registry-derived metrics, such as risk-adjusted PAL rates, drainage duration, and length of stay could further promote convergence toward best practice and stimulate targeted quality improvement initiatives.

Although drain strategy was associated with PAL and severe surgical emphysema, patient-level and procedural factors remained the dominant determinants of postoperative pulmonary complications, composite morbidity, unplanned ICU admission, and mortality. Smoking status, respiratory comorbidity, and performance status consistently predicted adverse outcomes, with current smokers and patients with chronic respiratory disease experiencing higher rates of prolonged air leak, postoperative pulmonary complications, and ICU use. Anatomic resections, systematic lymphadenectomy, and technical conversion were also independently associated with PAL and downstream morbidity, underscoring the influence of resection extent and intraoperative complexity.^21^

These observations support a model in which chest drain management modulates risk within the boundaries set by underlying patient vulnerability and procedural stress rather than acting as the primary driver of postoperative events. Optimizing drain strategy alone is therefore unlikely to abolish PAL or pulmonary complications but can reduce their frequency and mitigate their impact when combined with systematic risk assessment, prehabilitation, smoking cessation, and meticulous intraoperative technique.^21^ This integrated perspective aligns with ERAS principles, which emphasize multifaceted, pathway-based interventions rather than reliance on single technical modifications.

### Strengths and Limitations

This study has several strengths. The registry design was prospective and multicenter, included 21 high-volume thoracic units across multiple continents, and captured a large, contemporary cohort undergoing predominantly minimally invasive pulmonary resections. Detailed data on drain size, drain type, suction use, and adjunctive hemostatic measures were prospectively recorded alongside comprehensive patient, operative, and outcome variables, enabling robust multivariable adjustment and exploration of center-level effects. The use of both regression modelling and propensity score–matched analyses strengthens causal inference, particularly for comparisons of digital versus analogue drainage systems and different drain size strategies.

However, the limitations inherent to observational registry data must be acknowledged. Residual confounding by unmeasured factors, such as detailed leak morphology, surgeon-level preferences, or intraoperative lung-protection strategies, cannot be excluded despite adjustment for a wide range of covariates and the inclusion of center as a fixed effect. Pain, patient-reported outcomes, and granular mobilization metrics were not systematically collected, limiting our ability to balance the potential comfort advantages of smaller drains against the observed reduction in PAL with larger drains. Definitions and thresholds for PAL and drain removal, particularly in centers using analogue systems, may have varied subtly despite standardized registry fields, potentially contributing to inter-center differences. Event rates for in-hospital mortality and some less common complications were low, resulting in wide CIs and limiting the precision of effect estimates for these end points. Mortality follow-up was limited to the index hospitalization, because postdischarge and 90-day mortality were not systematically recorded across centers. As a result, we cannot exclude late deaths occurring after discharge that might be related to postoperative complications or chest drain management. Finally, cost data were not collected, precluding formal economic evaluation of digital drainage systems or specific drain strategies.

### Clinical Implications and Future Research

The present findings have several immediate clinical implications. First, they suggest that routine use of very small-bore drains after anatomic pulmonary resection may warrant reconsideration, particularly in higher-risk patients and complex resections, given the association of larger drains with reduced PAL and severe surgical emphysema without a signal for increased pulmonary complications or mortality. Second, they support broader adoption of digital drainage systems where feasible, not primarily as a means of reducing “true” PAL but as a tool for standardizing decision-making, enabling earlier safe drain removal, and improving the interpretability of air-leak data across centers. Third, they argue for explicit, pathway-based drain management protocols that integrate drain size, digital monitoring, and predefined removal thresholds, rather than relying on informal, center-specific customs.

Future research should focus on pragmatic, registry-embedded trials that randomize patients or centers to distinct drain strategies within routine care, leveraging existing data-collection infrastructure to efficiently evaluate outcomes at scale. Priority questions include the optimal range of drain caliber for different resection types and patient risk profiles, the comparative effectiveness and cost-effectiveness of universal versus selective use of digital systems, and the impact of harmonized, digitally informed definitions of PAL on both clinical decision-making and trial design. Incorporation of patient-reported pain, quality of life, and functional recovery metrics will be essential to balance physiological benefits against comfort and satisfaction, particularly when considering larger drains. Ultimately, integration of standardized, evidence-based drain pathways into ERAS programs and iterative benchmarking through multicenter registries may offer the most effective route to reducing unwarranted variation, improving outcomes, and optimizing resource use after pulmonary resection.

## Conflict of Interest Statement

The authors reported no conflicts of interest.

The *Journal* policy requires editors and reviewers to disclose conflicts of interest and to decline handling or reviewing manuscripts for which they may have a conflict of interest. The editors and reviewers of this article have no conflicts of interest.
