# Supplementary material for: Contemporary chest drain practice after pulmonary resection: A multicenter registry study of drain strategy, outcomes, and center-level variation
Source: JTCVS Open. 2026 Aug 13;33:102053. doi: 10.1016/j.xjon.2026.102053 (PMC13711205; doi:10.1016/j.xjon.2026.102053)
Supplement: Supplementary file 1 [file mmc1.docx]

**Supplementary Tables (S1-8)**

**S1** – A breakdown of the cardiovascular comorbidities

| **Cardiovascular Comorbidity** | **TOTAL** | **%** |
| --- | --- | --- |
| Atrial Fibrillation | **280** | 9.608785 |
| Ischaemic Heart Disease | **218** | 7.481126 |
| Pulmonary Hypertension | **20** | 0.686342 |
| Valvular Heart Disease | **7** | 0.24022 |
| Previous Cardiac Surgery | **59** | 2.024708 |
| Hypertension | **876** | 30.06177 |

**S2** – A breakdown of the respiratory comorbidities

| **Respiratory Comorbidity** | **TOTAL** | **%** |
| --- | --- | --- |
| COPD | **431** | 14.79067 |
| Asthma | **297** | 10.19218 |
| Bronchiectasis | **23** | 0.789293 |
| Interstitial Lung Disease | **87** | 2.985587 |
| Previous TB | **161** | 5.525051 |

**S3** – A breakdown of complication rates in entire cohort

| **Complication Tally** | **TOTAL** | **%** |
| --- | --- | --- |
| Pneumonia | **78** | 2.676733 |
| AF | **76** | 2.608099 |
| Peri-op MI | **12** | 0.411805 |
| DVT/PE | **56** | 1.872909 |
| Stroke | **5** | 0.171585 |
| Bowel obstruction | **3** | 0.102951 |
| Unplanned ICU admission | **25** | 0.857927 |
| Chylothorax | **17** | 0.583391 |
| Dysphonia | **16** | 0.549073 |
| BPF | **2** | 0.068634 |
| Atelectasis | **15** | 0.514756 |
| ARDS | **4** | 0.137268 |

**S4** – Management techniques for prolonged post-operative air leaks

| **Management of PAL** | **TOTAL** | **%** |
| --- | --- | --- |
| Conversative | 247 | 99.59677 |
| Blood pleurodesis | 24 | 9.677419 |
| Talc slurry | 9 | 3.629032 |
| Re-operation | 15 | 6.048387 |
| Endobronchial Valve | 11 | 4.435484 |

**S5** – A breakdown of the haemostatic adjuncts utilised

| **Haemostatic Adjuncts** | **TOTAL** | **%** |
| --- | --- | --- |
| **Matrix Agents** | **479** | **16.43789** |
| Arista | 291 | 9.986273 |
| Fibrillar | 180 | 6.177076 |
| Haemopatch | 8 | 0.274537 |
| **Biological Agents** | **391** | **13.41798** |
| Floseal/Surgiflo | 231 | 7.927248 |
| Tachosil | 107 | 3.671929 |
| Beriplast | 49 | 1.681537 |
| Bolheal | 3 | 0.102951 |
| Bioglue | 1 | 0.034317 |
| **Synthetic/Sealants** | **104** | **3.568977** |
| PGA sheet | 15 | 0.514756 |
| Veriset patch | 89 | 3.054221 |
| **Sutures** | **128** | **4.392588** |

# **S6** - Table 2. Centre-Level Practice Patterns and Outcomes

*Centres are anonymised and labelled alphabetically (A-U). PAL = prolonged air leak (>5 days); LOS = length of stay.*

| Centre | Sample Size (n) | Digital Drain Usage (%) | Median Drain Size (Fr) | PAL Rate (%) | Median LOS (days) | Median Drain Duration (days) |
| --- | --- | --- | --- | --- | --- | --- |
| A | 273 | 45 | 24 | 8.5 | 5 | 3 |
| B | 89 | 95 | 24 | 11.2 | 5 | 2 |
| C | 417 | 98 | 24 | 0.1 | 6 | 3 |
| D | 40 | 15 | 28 | 32.8 | 4 | 3 |
| E | 237 | 95 | 24 | 10.5 | 5 | 2 |
| F | 191 | 85 | 24 | 11.8 | 5 | 2 |
| G | 156 | 92 | 28 | 12.2 | 5 | 3 |
| H | 25 | 8 | 24 | 8.9 | 6 | 3 |
| I | 402 | 80 | 24 | 11.5 | 5 | 2 |
| J | 103 | 5 | 24 | 12.1 | 5 | 3 |
| K | 179 | 2 | 20 | 25.1 | 6 | 4 |
| L | 76 | 96 | 24 | 10.8 | 5 | 3 |
| M | 153 | 90 | 20 | 9.2 | 6 | 3 |
| N | 8 | 25 | 24 | 23.5 | 5 | 4 |
| O | 40 | 15 | 28 | 19.8 | 5 | 4 |
| P | 187 | 18 | 28 | 16.2 | 5 | 3 |
| Q | 153 | 2 | 24 | 8.8 | 6 | 3 |
| R | 83 | 3 | 28 | 11.2 | 5 | 3 |
| S | 105 | 20 | 28 | 16.5 | 5 | 3 |
| T | 274 | 92 | 20 | 8.2 | 5 | 2 |
| U | 157 | 5 | 28 | 10.8 | 5 | 3 |

| **Code** | **Centre** | **N** |
| --- | --- | --- |
| Centre A | Turin, Italy | 83 |
| Centre B | Bellinzona, Italy | 89 |
| Centre C | University Hospitals Birmingham, England | 417 |
| Centre D | Feldkirch, Austria | 40 |
| Centre E | Guy’s Hospital, London, England | 237 |
| Centre F | Hiroshima University Hospital, Japan | 191 |
| Centre G | Hôpitaux universitaires de Genève (Geneva University Hospitals) | 156 |
| Centre H | Hospital Universitario Puerta de Hierro (HUPH), Madrid, Spain | 25 |
| Centre I | Istituto Europeo di Oncologia – IEO, Milan | 402 |
| Centre J | IRCCS San Gerardo dei Tintori, Monza, Italy | 103 |
| Centre K | Kyorin University, Tokyo, Japan | 179 |
| Centre L | Linz, Austria | 76 |
| Centre M | Marseille, France | 153 |
| Centre N | Nancy, France | 8 |
| Centre O | Politecnico di Milano (PoliMi), Milan, Italy | 40 |
| Centre P | SCF, Rome, Italy | 187 |
| Centre Q | Shanghai East hospital, China | 153 |
| Centre R | Siena, Italy | 273 |
| Centre S | University Hospitals Southampton | 105 |
| Centre T | Ghent University Hospital, Belgium | 274 |
| Centre U | Inova Alexandria Hospital, Virginia, USA | 157 |

**S7** – Mapping of anonymised centre identifiers (Table 2 and Figures 1-2) to participating centres

**S8** - Univariable analysis of factors associated with prolonged air leak (PAL)

| **Variable** | **Level** | **No PAL** | **PAL** | **Total N** | **P value** |
| --- | --- | --- | --- | --- | --- |
| Age | Continuous | 69.0 (61.0-75.0) | 70.4 (61.7-76.0) | 2428 | **0.0302** |
| Sex | F | 1078 | 101 (8.6%) | 1179 | **0.0182** |
|  | M | 1105 | 144 (11.5%) | 1249 |  |
| BMI | Continuous | 24.7 (22.2-27.9) | 23.6 (21.0-26.6) | 2202 | **<0.0001** |
| Smoking status | Current Smoker | 423 | 79 (15.7%) | 502 | **<0.0001** |
|  | Ex-Smoker | 997 | 123 (11.0%) | 1120 |  |
|  | Never Smoker | 763 | 43 (5.3%) | 806 |  |
| ECOG PS | Continuous | 1.0 (0.0-1.0) | 1.0 (0.0-1.0) | 2428 | 0.8736 |
| Previous chest surgery | No | 1962 | 214 (9.8%) | 2176 | 0.2241 |
|  | Yes | 221 | 31 (12.3%) | 252 |  |
| Induction treatment | No | 2075 | 231 (10.0%) | 2306 | 0.5405 |
|  | Yes | 108 | 14 (11.5%) | 122 |  |
| Approach | Open | 259 | 40 (13.4%) | 299 | **0.0283** |
|  | RATS | 731 | 91 (11.1%) | 822 |  |
|  | VATS | 1193 | 114 (8.7%) | 1307 |  |
| Type of resection | Bilobectomy | 35 | 8 (18.6%) | 43 | **0.0002** |
|  | Lower lobectomy | 395 | 48 (10.8%) | 443 |  |
|  | Middle lobectomy | 115 | 9 (7.3%) | 124 |  |
|  | Segmentectomy (multiple) | 231 | 19 (7.6%) | 250 |  |
|  | Segmentectomy (single) | 273 | 29 (9.6%) | 302 |  |
|  | Upper lobectomy | 704 | 107 (13.2%) | 811 |  |
|  | Wedge | 430 | 25 (5.5%) | 455 |  |
| Lymphadenectomy | Not performed | 397 | 33 (7.7%) | 430 | **<0.0001** |
|  | Sampling | 509 | 31 (5.7%) | 540 |  |
|  | Systematic lymphadenectomy | 1277 | 181 (12.4%) | 1458 |  |
| Conversion | No | 2110 | 229 (9.8%) | 2339 | **0.0013** |
|  | Yes (bleeding) | 23 | 1 (4.2%) | 24 |  |
|  | Yes (technical) | 50 | 15 (23.1%) | 65 |  |
| Digital drain | No | 1439 | 134 (8.5%) | 1573 | **0.0007** |
|  | Yes | 744 | 111 (13.0%) | 855 |  |
| Drain size | 20F | 221 | 11 (4.7%) | 232 | **<0.0001** |
|  | 22F | 72 | 21 (22.6%) | 93 |  |
|  | 24F | 883 | 92 (9.4%) | 975 |  |
|  | 28F | 1007 | 121 (10.7%) | 1128 |  |
| Drain type | Coated | 13 | 2 (13.3%) | 15 | 0.7827 |
|  | PVC | 1163 | 123 (9.6%) | 1286 |  |
|  | Rocket | 1 | 0 (0.0%) | 1 |  |
|  | Silicone | 1006 | 120 (10.7%) | 1126 |  |
| Use of suction | -10 cmH2O | 1255 | 148 (10.5%) | 1403 | 0.4132 |
|  | -20 cmH2O | 928 | 97 (9.5%) | 1025 |  |
| Primary haemostasis | No | 1046 | 129 (11.0%) | 1175 | 0.1775 |
|  | Yes | 1137 | 116 (9.3%) | 1253 |  |
| Previous cardiovascular comorbidity | No | 1017 | 107 (9.5%) | 1124 | 0.4176 |
|  | Yes | 1166 | 138 (10.6%) | 1304 |  |
| Previous respiratory comorbidity | No | 1504 | 128 (7.8%) | 1632 | **<0.0001** |
|  | Yes | 679 | 117 (14.7%) | 796 |  |

*Continuous variables are presented as median (IQR); categorical variables are presented as counts with PAL percentages.*

**S9** - Relationship between drainage volume and duration of drainage. First 24‑hour and total drainage volumes are shown by quintile with corresponding median drain duration and interquartile range. Early (24‑hour) output shows only a weak association with duration, whereas total cumulative output demonstrates a clear stepwise increase in median days of drainage.

| **Quintile of drainage volume** | **Median drainage volume (ml)** | **Median drain duration (days)** | **IQR (days)** |
| --- | --- | --- | --- |
| First 24‑hour drainage Q1 | 65 | 2 | 1–3 |
| First 24‑hour drainage Q2 | 140 | 2 | 1–4 |
| First 24‑hour drainage Q3 | 200 | 3 | 2–5 |
| First 24‑hour drainage Q4 | 300 | 3 | 2–5 |
| First 24‑hour drainage Q5 | 475 | 3 | 2–6 |
| Total drainage Q1 | 100 | 1 | 1–3 |
| Total drainage Q2 | 250 | 2 | 1–4 |
| Total drainage Q3 | 500 | 3 | 2–5 |
| Total drainage Q4 | 1000 | 4 | 3–6 |
| Total drainage Q5 | 1500 | 5 | 4–7 |
